# Therapeutic Targets in Glioblastoma: Molecular Pathways, Emerging Strategies, and Future Directions

**DOI:** 10.3390/cells14070494

**Published:** 2025-03-26

**Authors:** Justin Tang, Nishika Karbhari, Jian L. Campian

**Affiliations:** 1 Department of Biomedical Science, University of Guelph, Guelph, ON N1G 2W1, Canada; 2 Department of Oncology, Mayo Clinic, Rochester, MN 55905, USA; karbhari.nishika@mayo.edu (N.K.); campian.jian@mayo.edu (J.L.C.)

**Keywords:** glioblastoma, molecular pathways, targeted therapy, immunotherapy, epigenetic modulation, tumor microenvironment, blood–brain barrier, nanotechnology, CAR T cell therapy, oncolytic viruses, MGMT promoter methylation, gene therapy, combination therapies, biomarkers, drug delivery systems

## Abstract

Glioblastoma (GBM) is the most aggressive primary brain tumor in adults, characterized by rapid growth, invasive infiltration into surrounding brain tissue, and resistance to conventional therapies. Despite advancements in surgery, radiotherapy, and chemotherapy, median survival remains approximately 15 months, underscoring the urgent need for innovative treatments. Key considerations informing treatment development include oncogenic genetic and epigenetic alterations that may dually serve as therapeutic targets and facilitate treatment resistance. Various immunotherapeutic strategies have been explored and continue to be refined for their anti-tumor potential. Technical aspects of drug delivery and blood–brain barrier (BBB) penetration have been addressed through novel vehicles and techniques including the incorporation of nanotechnology. Molecular profiling has emerged as an important tool to individualize treatment where applicable, and to identify patient populations with the most drug sensitivity. The goal of this review is to describe the spectrum of potential GBM therapeutic targets, and to provide an overview of key trial outcomes. Altogether, the progress of clinical and preclinical work must be critically evaluated in order to develop therapies for GBM with the strongest therapeutic efficacy.

## 1. Introduction

GBM, classified by the World Health Organization (WHO) as an IDH wild-type grade 4 astrocytoma, is the most aggressive primary brain tumor in adults [1]. Originating from glial cells, GBMs are characterized by rapid proliferation, diffuse infiltration into surrounding brain tissue, extensive angiogenesis, and pronounced genomic instability [2]. The heterogeneous nature of these tumors encompasses a wide spectrum of genetic, epigenetic, and phenotypic variations among tumor cells, contributing to their complex biology and resistance to therapy [3]. Originating from glial cells, glioblastomas are characterized by rapid proliferation, diffuse infiltration into surrounding brain tissue, extensive angiogenesis, and pronounced genomic instability [2]. The heterogeneous nature of these tumors encompasses a wide spectrum of genetic, epigenetic, and phenotypic variations among tumor cells, contributing to their complex biology and resistance to therapy [3]. The term “multiforme” has been largely omitted in the recent literature to reflect a more precise understanding of the tumor’s characteristics and to avoid confusion [4].

Globally, glioblastoma accounts for approximately 15% of all primary brain and central nervous system (CNS) tumors and about 45% of malignant brain tumors [5]. The annual incidence rate is approximately 3.2 per 100,000 population, making it the most common primary malignant brain tumor in adults [6]. Glioblastoma predominantly affects individuals between the ages of 45 and 70, with a slight male predominance [7]. Epidemiological studies have shown variations in incidence based on geographic location, ethnicity, and environmental factors, but the underlying reasons for these differences remain under investigation [8].

Patients with glioblastoma often present with symptoms that reflect the tumor’s location and the resultant mass effect on adjacent brain structures [9]. Common clinical manifestations include persistent headaches, seizures, focal neurological deficits such as weakness or sensory disturbances, and cognitive or behavioral changes [10]. Increased intracranial pressure may lead to nausea, vomiting, and papilledema [11]. Diagnosis typically involves neuroimaging studies, with magnetic resonance imaging (MRI) being the gold standard [12]. MRI with contrast enhancement reveals a heterogeneous mass with irregular borders, central necrosis, and peripheral edema [13]. Advanced imaging techniques like diffusion tensor imaging and magnetic resonance spectroscopy can provide additional insights into tumor characteristics [14]. Definitive diagnosis requires histopathological examination obtained via stereotactic biopsy or surgical resection [15]. Molecular profiling is increasingly used to identify genetic alterations that can inform prognosis and therapeutic strategies [16].

The prognosis for glioblastoma remains poor despite aggressive treatment. The median overall survival is approximately 14 to 16 months following standard therapy, with a two-year survival rate of about 26% and a five-year survival rate less than 10% [17]. Factors influencing prognosis include patient age, performance status, the extent of surgical resection, and molecular markers such as O6-methylguanine-DNA methyltransferase (MGMT) promoter methylation status and isocitrate dehydrogenase (IDH) mutation status [18]. Younger patients with good performance status and favorable molecular profiles tend to have better outcomes [19].

The current standard of care for glioblastoma involves maximal safe surgical resection, followed by radiotherapy and chemotherapy [17]. Maximal safe surgical resection is the cornerstone of glioblastoma management, aiming to remove as much of the tumor mass as possible without compromising neurological function [20]. Extensive resection reduces tumor burden, alleviates symptoms, and enhances the effectiveness of adjuvant therapies [21]. Techniques such as intraoperative MRI and fluorescence-guided surgery using 5-aminolevulinic acid have improved the extent of resection [22]. However, complete removal is often unattainable due to the tumor’s infiltrative nature and proximity to eloquent brain regions [23]. Postoperative radiotherapy is administered to target residual tumor cells and delay recurrence [24]. Conventional fractionated external beam radiation therapy delivers a total dose of 60 Gy over six weeks [25]. Radiotherapy has been shown to improve median survival by several months compared to surgery alone [24]. Challenges include the radioresistant nature of glioblastoma cells, the potential for radiation-induced neurotoxicity, and the need to spare healthy brain tissue to preserve function [26]. Temozolomide, an oral alkylating agent, has become the standard chemotherapeutic agent for glioblastoma [17]. It induces DNA damage by methylating guanine residues, leading to tumor cell apoptosis [27]. The Stupp protocol, which combines concurrent temozolomide with radiotherapy followed by six cycles of adjuvant temozolomide, has demonstrated a survival benefit [17]. However, temozolomide’s efficacy is limited by the tumor’s intrinsic resistance mechanisms and systemic toxicity, including myelosuppression [28].

This review aims to explore emerging therapeutic targets in glioblastoma by examining the molecular pathways and cellular processes that drive tumor progression and resistance. By understanding these mechanisms, novel targets for intervention can be identified. The focus will be on molecular pathogenesis, key signaling pathways, tumor microenvironment interactions, and innovative therapeutic approaches under investigation.

## 2. Molecular Pathogenesis of GBM

The distinct molecular profile of GBM contributes to disease pathogenesis. Molecular-level alterations can also serve as important therapeutic targets. GBM is characterized by a complex array of genetic mutations and epigenetic modifications that drive its aggressive phenotype. Key genetic alterations include mutations in TP53, PTEN, and EGFR, each contributing to the disruption of critical cellular processes [26]. TP53 is a key regulator of genomic stability. TP53 mutations, observed in approximately 31–38% of cases, impair DNA repair, cell cycle arrest, and apoptosis [27]. These mutations are more common in secondary GBMs, but also occur in primary GBMs [28]. PTEN mutations, present in 24–37% of GBMs, are mainly present in primary GBMs and lead to unchecked activation of the PI3K/AKT signaling pathway, promoting cell growth and survival by inhibiting apoptosis [29]. EGFR amplification and mutations are observed in 36–60% of primary GBMs, with the EGFRvIII variant present in 20–50% of EGFR-amplified cases [30]. This variant results from a deletion in the extracellular domain and produces constitutive receptor activation, leading to downstream proliferation, survival, and angiogenesis [31,32]. Mutations are detailed in Table 1.

Epigenetic changes, such as DNA methylation and histone modifications, play a crucial role in GBM pathogenesis [33,34,35,36]. Methylation of the MGMT promoter reduces the expression of the MGMT enzyme responsible for repairing alkylated DNA [37,38]. Patients with MGMT promoter methylation accordingly exhibit increased sensitivity to the alkylating chemotherapy temozolomide.

GBM cells exploit several signaling pathways to support their malignant behavior, providing opportunities for targeted therapeutic interventions [39,40,41]. The PI3K/Akt/mTOR pathway is central to regulating cell growth, survival, metabolism, and angiogenesis [42]. Activation occurs through receptor tyrosine kinases (RTKs) like EGFR and PDGFR [43]. In GBM, aberrant activation of this pathway is common due to genetic alterations in PTEN, PIK3CA, or amplification of RTKs, leading to increased protein synthesis, inhibition of apoptosis, and promotion of cell cycle progression [44]. The mitogen-activated protein kinase (MAPK) pathway is another critical signaling cascade involved in cell proliferation and differentiation [45]. While mutations leading to constitutive activation of this pathway are less common in GBM, they can result from upstream RTK activation, and cross-talk between the PI3K/Akt/mTOR and MAPK pathways contributes to tumor growth and resistance mechanisms [46].

**Figure 1:**
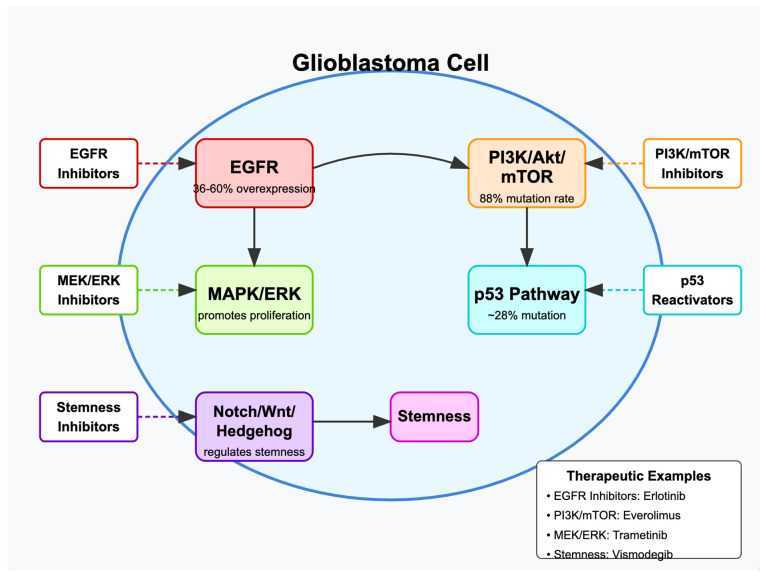
Key molecular pathways in glioblastoma and their therapeutic targets.

Additionally, developmental signaling pathways such as Notch, Wnt/β-catenin, and Hedgehog are implicated in maintaining cancer stem cells (CSCs) within GBM, which contribute to tumor development, resistance to therapy, and recurrence [47]. The Notch pathway promotes cell survival and self-renewal, supporting the maintenance of CSCs when overactivated in GBM [48]. Dysregulation of the Wnt/β-catenin pathway leads to increased β-catenin levels and transcription of oncogenic targets [49], while the Hedgehog pathway influences stem cell maintenance and has been associated with GBM aggressiveness [50]. Figure 1 and Table 2 provides an overview of some key signaling pathways in GBM and associated targeted therapies.

The GBM tumor microenvironment (TME) also plays an important role in disease pathogenesis. The TME contains a complex network of cellular, molecular, and biochemical interactions that can facilitate tumor growth and resistance to therapy [51], but also shape many of the key signaling pathways implicated in GBM progression. Consequently, understanding how the TME influences aberrant signaling within tumor cells is essential for identifying effective therapeutic targets. Hypoxia within tumors results from rapid cell proliferation outpacing new blood vessel development [52]. Stabilization of hypoxia-inducible factors (HIFs) under low-oxygen conditions leads to upregulation of the vascular endothelial growth factor (VEGF), stimulating angiogenesis and creating abnormal, leaky vasculature that contributes to tumor growth and invasion [53]. GBM also alters the TME composition through immunoevasive strategies, including the secretion of immunosuppressive cytokines like transforming growth factor-beta (TGF-β) and interleukin-10 (IL-10) [54], upregulation of programmed death-ligand 1 (PD-L1) on tumor cells to inhibit T cell function [55], and recruitment of regulatory T cells (Tregs) and myeloid-derived suppressor cells (MDSCs) to suppress anti-tumor immunity [56]. Interactions with surrounding stroma support tumor growth. Astrocytes can provide metabolic support and survival factors [57], microglia and macrophages can be co-opted to a tumor-promoting phenotype [58], and matrix metalloproteinases (MMPs) can degrade the extracellular matrix to facilitate invasion [59].

## 3. Emerging Therapeutic Targets Under Investigation

### 3.1. Targeting Growth Factor Receptors

Targeting growth factor receptors, such as the epidermal growth factor receptor (EGFR), has been an important focus of investigation due to the relatively high incidence of alterations in GBM [30]. Monoclonal antibodies, like cetuximab, are designed to bind to the extracellular domain of EGFR, blocking ligand binding and receptor activation [60]. However, their efficacy is limited by effective BBB penetration and the heterogeneity of EGFR mutations across different tumor regions [61]. Small molecule tyrosine kinase inhibitors, like erlotinib, inhibit EGFR activity by competing with ATP binding [62]. However, clinical trials have shown limited success due to insufficient central nervous system (CNS) drug delivery and resistance mechanisms, like PTEN loss [45,63].

### 3.2. Signal Transduction Pathway Inhibitors

Inhibitors targeting the frequently activated PI3K/Akt/mTOR pathway are under exploration [42]. Agents such as rapamycin analogs (e.g., everolimus) can reduce cell proliferation and induce autophagy [64]. However, their clinical efficacy is often limited due to feedback activation loops and incomplete pathway inhibition [65]. To overcome resistance mechanisms, dual PI3K/mTOR inhibitors are being studied [66]. MEK and ERK inhibitors, like trametinib, are also under evaluation, particularly in tumors with specific mutations or as part of combination therapies [67].

### 3.3. Epigenetic Modulators

Epigenetic modulators have emerged as promising therapeutic agents in GBM treatment [37]. Histone deacetylase (HDAC) inhibitors, like vorinostat, modify chromatin structure to alter gene expression, reactivating tumor suppressor genes and inducing apoptosis [68]. These agents are attractive due to their ability to cross the BBB [69]. DNA methyltransferase (DNMT) inhibitors such as azacitidine aim to demethylate DNA and restore normal gene function [70]. Clinical trials are ongoing to determine their efficacy and safety in patients with GBM.

### 3.4. Immunotherapy

Immunotherapy represents a rapidly evolving frontier in GBM treatment. While no immunotherapy has achieved regulatory approval for GBM to date, numerous approaches are under active investigation, including immune checkpoint inhibitors (ICIs), chimeric antigen receptor (CAR) T cell therapies, therapeutic vaccines, oncolytic viruses, cytokine-based strategies, and agents targeting the immunosuppressive tumor microenvironment, such as tumor-associated macrophages (TAMs) [71].

### 3.5. Immune Checkpoint Inhibitors (ICIs)

The immunosuppressive tumor microenvironment of GBM blunts effective antitumor immune responses by various mechanisms, including the upregulation of immune checkpoints. Common inhibitory receptors such as programmed cell death-1 (PD-1), programmed cell death-ligand 1 (PD-L1), cytotoxic T-lymphocyte-associated protein 4 (CTLA-4), lymphocyte-activation gene 3 (LAG-3), and T cell immunoglobulin and mucin-domain containing-3 (TIM-3) play pivotal roles in dampening T cell activity within GBM [72].

#### 3.5.1. PD-1/PD-L1 Blockade

Agents like nivolumab and pembrolizumab block PD-1, restoring T cell function and potentially improving tumor cell clearance. Despite encouraging efficacy in other solid tumors, trials in GBM have been disappointing. For example, phase III trials combining nivolumab with standard therapy did not improve overall survival compared to historical controls. The lack of success highlights the formidable immunosuppressive milieu and the necessity of careful patient selection, rational combination therapies, and novel trial designs [73].

#### 3.5.2. CTLA-4 Inhibition

Ipilimumab, a CTLA-4 blocking antibody, has shown limited efficacy in GBM. Unlike the PD-1 blockade, CTLA-4 inhibition often leads to more global immune activation and higher rates of immune-related adverse events. Combinations of PD-1 and CTLA-4 inhibitors, though potentially more efficacious, also face toxicity and tolerability challenges [74].

#### 3.5.3. Next-Generation Checkpoints (LAG-3, TIM-3, and Others)

Targeting emerging checkpoints, including LAG-3, TIM-3, and others currently under exploration (e.g., TIGIT, VISTA), may overcome resistance to PD-1/CTLA-4 blockade. Early-phase trials are ongoing, evaluating whether simultaneous blockade of multiple inhibitory receptors can more effectively penetrate GBM’s robust immune defenses [75].

### 3.6. Chimeric Antigen Receptor (CAR) T Cell Therapy

CAR T cell therapies genetically engineer patient-derived T cells to recognize specific antigens on GBM cells. The success of CAR T cells in hematological malignancies has spurred interest in solid tumors, including GBM.

#### Established Targets

Early CAR T cell trials focused on EGFR variant III (EGFRvIII), which is frequently mutated in GBM, as well as on interleukin-13 receptor α2 (IL13Rα2). While clinical responses have been observed, durable remissions are rare, likely due to heterogeneous antigen expression, T cell exhaustion, and the highly immunosuppressive GBM microenvironment [76,77].

### 3.7. Vaccines and Peptide-Based Immunotherapies Under Investigation

Vaccines aim to induce or enhance an endogenous, tumor-specific immune response.

#### 3.7.1. Peptide-Based Vaccines

Peptide vaccines targeting tumor-associated antigens (TAAs) or neoantigens unique to GBM cells (e.g., EGFRvIII) represent a promising strategy. By focusing on mutations not found in normal tissue, these vaccines minimize the risk of off-target effects and maximize tumor specificity [78].

#### 3.7.2. Dendritic Cell (DC) Vaccines

DC vaccines involve loading patient-derived DCs with tumor peptides, lysates, or mRNA to present TAAs to T cells. Clinical trials have shown that dendritic cell (DC) vaccines can induce robust immune responses and may prolong survival in select patient populations [79]. Notably, a recent phase 3 prospective, externally controlled trial of an autologous tumor lysate-loaded DC vaccine (DCVax-L) in newly diagnosed and recurrent glioblastoma reported significantly improved median overall survival compared to matched external controls. In newly diagnosed patients, median overall survival was extended to 19.3 months from randomization (22.4 months from surgery), compared to 16.5 months in controls, and in recurrent disease median overall survival was 13.2 months vs. 7.8 months in controls. These findings highlight the potential of DC-based immunotherapy to improve outcomes in malignant brain tumors [79]. Ongoing research optimizes antigen selection, DC maturation protocols, and combination strategies with ICIs or radiotherapy to enhance vaccine efficacy.

#### 3.7.3. Cell-Penetrating and Tumor-Targeting Peptides

Beyond classic vaccines, tumor-targeting peptides can selectively bind receptors overexpressed on GBM cells, serving as vehicles for diagnostics or targeted drug delivery. Cell-penetrating peptides (CPPs) offer an avenue for enhancing drug or gene therapy delivery directly into malignant cells, potentially improving therapeutic index [80].

### 3.8. Oncolytic Virus Therapies

Oncolytic viruses (OVs) are engineered to preferentially infect, replicate within, and lyse tumor cells. This not only causes direct oncolysis, but also exposes TAAs to the immune system, potentially converting an immunosuppressive “cold” tumor into an “immunologically hot” one.

#### 3.8.1. Virus Platforms

Genetically modified herpes simplex viruses (e.g., G207, G47Δ), adenoviruses (e.g., DNX-2401), and poliovirus derivatives (e.g., PVSRIPO) have demonstrated safety and suggested efficacy in early-phase clinical trials [81].

#### 3.8.2. Mechanistic Synergies

**Figure 2:**
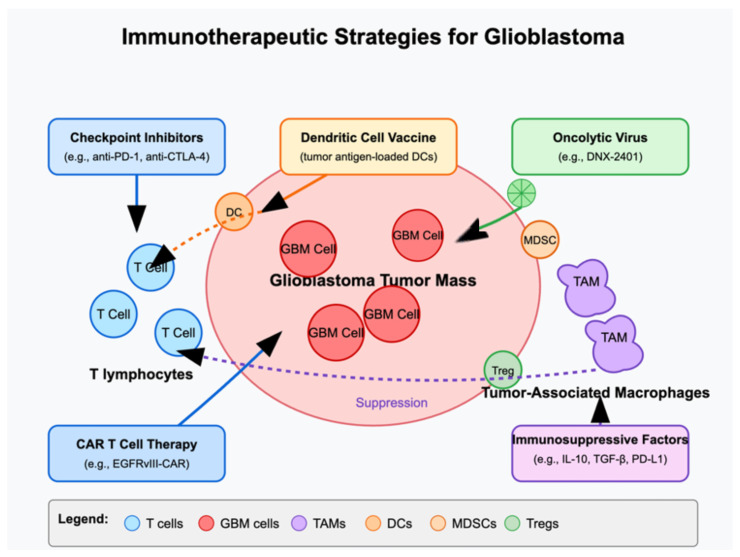
Overview of immunotherapy approaches in glioblastoma.

OVs can be combined with ICIs or CAR T cells to enhance antitumor immunity. As OVs disrupt the tumor extracellular matrix and local immunosuppression, T cells and immune effector cells may gain improved access to cancer cells, leading to synergistic therapeutic effects [82]. Key strategies have been summarized in Figure 2.

### 3.9. Cytokine-Based Therapies

Cytokines play a pivotal role in shaping the immune system’s ability to identify and eradicate tumor cells by modulating various cellular pathways that govern immune cell activation, proliferation, and effector functions [83]. Interleukin-2 (IL-2), for example, is a potent growth factor for T lymphocytes, driving their clonal expansion and augmenting their capacity to target malignancies. IL-2 not only increases the proliferation of cytotoxic T cells and helps sustain memory T cell populations, but also influences the differentiation of specialized T cell subsets that can release additional tumoricidal factors [83]. At the molecular level, IL-2 signaling is transduced through the IL-2 receptor complex, triggering downstream cascades such as the JAK/STAT pathway, which amplifies T cell responses against tumor-associated antigens [83]. However, IL-2 therapy must be carefully managed to avoid severe side effects, including vascular leak syndrome and the potential expansion of regulatory T cells that may dampen antitumor immunity [83].

In parallel, interferon-alpha (IFN-α) exerts broad antiproliferative and immunomodulatory effects that can be especially relevant for tumor suppression in the central nervous system (CNS). By binding to its cognate receptor, IFN-α activates transcription factors that upregulate major histocompatibility complex (MHC) expression, thereby enhancing the presentation of tumor antigens to T cells. IFN-α can also inhibit tumor cell replication through direct antiproliferative signaling, and can bolster immune surveillance by increasing the functional activity of natural killer cells [83]. Despite these promising mechanisms, the systemic delivery of IFN-α poses significant toxicity risks, which are further compounded by the difficulties of achieving effective cytokine concentrations within the CNS [64,73,83].

To address these challenges, investigational strategies focus on localized administration, such as intratumoral injection or convection-enhanced delivery, to circumvent systemic toxicity [83]. Moreover, engineered cytokine variants with selective receptor affinity are under development to direct immune activation more precisely toward tumor sites [83]. In combination with other immunotherapeutic modalities, such as immune checkpoint inhibitors, these refined cytokine approaches aim to strengthen antitumor responses while minimizing off-target effects, ultimately improving the potential for effective tumor clearance within the CNS [83].

### 3.10. Targeting the Tumor Microenvironment: Tumor-Associated Macrophages (TAMs)

GBM is characterized by a highly immunosuppressive microenvironment containing abundant TAMs, often skewed towards an M2-like, pro-tumorigenic phenotype that promotes angiogenesis, invasion, and resistance to therapy. Colony-stimulating factor-1 receptor (CSF-1R) inhibitors (e.g., PLX3397) target macrophage survival and polarization. Reducing the population of M2-like macrophages or reprogramming them towards an M1-like, antitumor state can enhance the efficacy of T cell-based therapies and improve patient outcomes [84]. Strategies to combine TAM-targeting agents with CAR T cells, vaccines, or ICIs may yield synergistic effects, altering the overall tumor ecosystem to favor an effective antitumor immune response.

### 3.11. Targeting Tumor Metabolism

Targeting tumor metabolism offers a new therapeutic avenue. Glutaminolysis, the process by which glutamine is converted into glutamate and subsequently into α-ketoglutarate in the tricarboxylic acid (TCA) cycle, supports the bioenergetic and biosynthetic needs of rapidly proliferating tumor cells. Inhibiting glutaminase (GLS), the enzyme catalyzing the first step of glutaminolysis, has shown potential in suppressing tumor growth. Studies indicate that GBM tissues can be categorized into glycolytic-dominant and mitochondrial-dominant types, with the latter also being glutaminolysis-dominant. Therefore, targeting the glutaminolysis pathway may be particularly effective for mitochondrial-dominant GBMs [85]. Additionally, metabolic reprogramming in GBM involves alterations in lipid metabolism, which contribute to tumor growth and survival. Targeting enzymes involved in fatty acid synthesis and oxidation pathways offers another avenue for therapeutic intervention. For instance, inhibitors of fatty acid synthase (FASN) have demonstrated efficacy in preclinical models by disrupting lipid biosynthesis essential for tumor cell membranes and signaling molecules. The researchers observed that treating GSCs with 20 μM cerulenin, a FASN inhibitor, led to a significant reduction in cell proliferation and invasiveness. Specifically, de novo lipogenesis decreased by approximately 40%, and the invasiveness of GSCs was reduced by 40–50% following cerulenin treatment. Additionally, the expression of stemness markers such as nestin, Sox2, and FABP7 decreased, while the differentiation marker GFAP increased [86]. Furthermore, ketogenic metabolic therapy (KMT) has been proposed as a potential treatment strategy for GBM. KMT aims to exploit the metabolic flexibility of GBM cells by restricting glucose availability and providing ketone bodies as alternative energy sources, thereby inhibiting glycolysis and glutaminolysis pathways. This approach may enhance the efficacy of existing treatments and improve patient outcomes [87].

GBM cells exhibit high glycolytic rates, leading to increased lactate production. Monocarboxylate transporters (MCTs) facilitate the export of lactate from tumor cells, maintaining intracellular pH balance and supporting continued glycolysis. Inhibiting MCTs can disrupt this process, leading to intracellular acidification and reduced tumor growth. Research has shown that targeting MCTs with inhibitors like α-cyano-4-hydroxycinnamic acid (CHC) effectively impairs GBM cell proliferation [88]. Also, isoform 2 of pyruvate kinase (PKM2) is a glycolytic enzyme that plays a pivotal role in tumor metabolism by regulating the final step of glycolysis. In cancer cells, PKM2 expression promotes aerobic glycolysis and supports anabolic processes essential for rapid cell proliferation. Silencing PKM2 increases apoptosis and promotes differentiation in both rat and human glioma spheroids. Mechanistically, PKM2 interacts with Oct4, a pivotal regulator of self-renewal and differentiation in stem cells, and this interaction influences glioma stemness. Treatment with the pyruvate dehydrogenase kinase inhibitor dichloroacetate (DCA) augments the formation of PKM2/Oct4 complexes, thereby inhibiting Oct4-dependent gene expression. Taken together, these findings highlight a molecular pathway in which PKM2 governs gliomagenesis by regulating stemness via Oct4, underlining the therapeutic potential of targeting PKM2 to disrupt cancer cell metabolism and tumor growth [89,90].

### 3.12. Bypassing the Blood–Brain Barrier

Despite these promising metabolic targets, effective treatment of GBM also requires overcoming a major hurdle in neuro-oncology: the restrictive nature of the blood–brain barrier (BBB). Focused ultrasound (FUS), combined with circulating microbubbles, has been developed to temporarily disrupt the BBB, allowing an enhanced delivery of therapeutic agents into the brain. This method has shown promise in preclinical models, and is currently being tested in clinical trials. For instance, low-intensity FUS with microbubbles can increase the intracranial concentration of chemotherapeutic agents, leading to significant tumor volume reduction and extended survival in patient-derived xenograft models. In situ and intranasal delivery of therapeutics are other approaches to bypass the BBB [91]. Convection-enhanced delivery (CED) allows for the direct infusion of drugs into the tumor site [91,92], while the intranasal route offers a non-invasive method to deliver drugs directly to the CNS via the olfactory and trigeminal nerves [93].

### 3.13. Drug Repurposing and Combination Therapies

Drug repurposing involves using existing drugs with known safety profiles for new therapeutic indications [94]. Agents like metformin and statins have shown potential in inhibiting GBM cell proliferation and inducing apoptosis [95]. Combination therapies that target multiple pathways simultaneously are being explored to overcome resistance mechanisms [96]. For example, combining metformin with temozolomide has demonstrated effectiveness in enhancing the chemotherapeutic response [97].

### 3.14. Oncolytic Viruses and Gene-Based Approaches

Gene therapy holds considerable promise for the treatment of glioblastoma (GBM), primarily by overcoming the barriers that currently limit effective drug delivery, particularly the blood–brain barrier (BBB). Rather than relying solely on conventional chemotherapeutics, gene therapy leverages the introduction of specific genetic sequences and regulatory factors into target cells to induce therapeutic outcomes [98]. This approach is especially relevant for GBM because of its infiltrative growth pattern and the BBB’s highly selective permeability, which severely restricts many systemically administered agents. By using delivery vectors, ranging from viral vectors engineered for tumor specificity to nonviral methods employing nanoparticles, gene therapy can not only bypass the BBB, but also enable highly targeted modulation of tumor and stromal cells [99]. This level of precision offers the possibility of minimizing systemic toxicity while maximizing therapeutic benefit in the central nervous system. Within the scope of gene therapy for GBM, oncolytic viruses (OVs) have garnered substantial interest due to their distinctive capacity to both infect and lyse tumor cells while simultaneously provoking a robust immune response. Their dual functionality addresses two of the most pressing issues in glioma therapy: achieving direct tumor cell killing and counteracting the profoundly immunosuppressive tumor microenvironment (TME). Although OVs were initially pursued for their cytolytic potential, whereby they selectively infect and replicate within tumor cells, ultimately causing cell death and tumor debulking, accumulating data suggest that their immunostimulatory attributes may hold equal or greater therapeutic importance. When OVs replicate within cancer cells, they can induce immunogenic cell death, which is characterized by the release of tumor-associated antigens (TAAs) in an inflammatory milieu. This environment spurs the activation of antigen-presenting cells (APCs), especially dendritic cells, which process and present these TAAs to T cells [98,99,100]. In turn, T cells become primed and activated, establishing a multifaceted immune assault that not only targets virus-infected tumor cells, but may also eradicate uninfected tumor cells bearing related antigens. As such, the immunologic bystander effect broadens the therapeutic impact beyond the cells directly lysed by the virus. Ultimately, this cascade reprograms the TME from an immunosuppressive setting, with limited T cell infiltration and high levels of inhibitory cytokines, into one that is more immunologically reactive and supportive of long-term tumor control [98,99,100].

**Figure 3:**
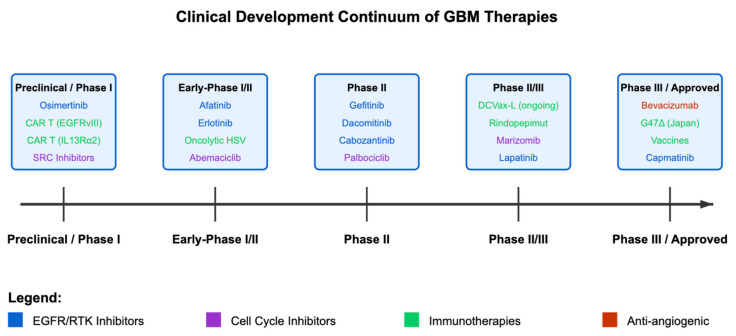
Overview of drugs and their respective phases.

A critical aspect of this transformation is the bridging of innate and adaptive immunity. OVs initiate a strong innate immune response, as pattern recognition receptors (PRRs) on cells such as macrophages and dendritic cells sense the viral particles. This early response can lead to the secretion of type I interferons and other cytokines that drive the recruitment of natural killer (NK) cells and other innate immune effectors. Subsequent antigen presentation to T cells lays the foundation for robust adaptive immunity that can persist beyond the presence of the virus itself. In many respects, OVs can be viewed as in situ vaccines, catalyzing antigen presentation and T cell priming directly within the tumor. This vaccination effect has the potential to establish long-lasting immunologic memory, which is crucial for preventing tumor recurrence, a major problem in GBM, where even small clusters of residual tumor cells can reignite aggressive growth [100,101]. The timeline of the GBM therapies are demonstrated in Figure 3.

Additionally, the use of oncolytic viruses as vectors allows for further genetic engineering, enhancing both tumor selectivity and immunomodulatory capacity. For instance, OVs can be modified to express immunostimulatory molecules, such as granulocyte-macrophage colony-stimulating factor (GM-CSF), or other cytokines that promote dendritic cell maturation and T cell expansion. This capacity for “arming” viruses with therapeutic transgenes is particularly attractive for GBM, as it addresses the need to overcome immune tolerance in the brain, a site that normally discourages excessive inflammation. Moreover, by engineering the viruses to replicate preferentially in tumor cells, healthy tissue exposure is minimized, thus improving the safety profile of oncolytic virotherapy. Among the diverse family of OVs under investigation, adenoviruses and herpes simplex viruses have shown especially promising outcomes in both preclinical and clinical settings [101]. Major GBM therapeutic targets have been detailed in Table 3, Table 4, Table 5 and Table 6.

### 3.15. Nanotechnology and Drug Delivery Systems

Nanoparticles are colloidal particles ranging from 1 to 100 nanometers in size, designed to carry drugs, genes, or imaging agents [101,102]. Their small size allows for enhanced permeation and retention within tumor tissues due to the leaky vasculature characteristic of GBM [87]. Researchers at Yale and the University of Connecticut have developed bioadhesive nanoparticles that adhere to tumor sites, enabling sustained and localized drug release. For instance, a study from Yale and the University of Connecticut introduced nanoparticles that, upon adhering to GBM tissues, gradually release therapeutic agents, enhancing treatment precision and minimizing systemic side effects [103]. Piezoelectric nanoparticles, such as barium titanate nanoparticles (BTNPs), have been investigated for their ability to generate electric stimulation upon exposure to ultrasound. Functionalized with antibodies targeting GBM cells, these nanoparticles can induce anti-proliferative effects and enhance sensitivity to chemotherapy. In vitro studies have demonstrated that ultrasound-mediated piezo-stimulation using BTNPs can significantly reduce GBM cell proliferation and promote apoptosis [104].

Exosome-like nanovesicles (ELNs) have been engineered to mimic natural exosome properties, serving as biocompatible carriers for drug delivery. These synthetic vesicles can be tailored to deliver therapeutic oligonucleotides, proteins, or chemotherapeutic agents directly to GBM cells, potentially enhancing treatment specificity and reducing off-target effects [105]. Research has shown that brain-targeted ELNs loaded with therapeutic oligonucleotides can elicit anti-tumor effects in GBM animal models [106]. Furthermore, marine-derived compounds have been utilized to create nanocarriers for drug delivery in GBM treatment. These nanocarriers offer biocompatibility and the ability to encapsulate a variety of therapeutic agents. Recent research has highlighted the potential of these systems to enhance drug delivery efficiency and therapeutic outcomes in GBM models [107].

Innovative DNA-based nanostructures, such as DNA nanotubes, have been engineered to deliver therapeutics directly to GBM tumors. These nanotubes can be functionalized with targeting ligands and therapeutic agents, facilitating precise delivery. Studies have shown that DNA nanotubes can effectively penetrate tumor tissues and deliver payloads, inhibiting tumor growth in experimental models [108]. Advancements in nanotechnology have facilitated the development of nanocarrier systems for gene therapy applications in GBM. These systems are designed to deliver genetic material, such as siRNA or plasmid DNA, to tumor cells, modulating gene expression to inhibit tumor growth. Recent studies have demonstrated the potential of these nanocarriers to enhance the efficacy of gene therapies in GBM treatment [109].

Surface modifications can exploit endogenous transport mechanisms across the BBB; for instance, coating nanoparticles with ligands targeting transferrin receptors or low-density lipoprotein receptors facilitates receptor-mediated transcytosis into the CNS [110]. nanoparticles employed in GBM research include liposomes, solid lipid nanoparticles, dendrimers, and polymeric nanoparticles. Liposomal formulations can encapsulate chemotherapeutic agents like temozolomide or doxorubicin, protecting them from degradation and enhancing CNS penetration [111]. Additionally, nanoparticles can be loaded with multiple agents, facilitating the delivery of combination therapies that target different tumor pathways simultaneously [96]. Magnetic nanoparticles offer dual functions of drug delivery and diagnostic imaging [112]. Superparamagnetic iron oxide nanoparticles can be guided to the tumor site using external magnetic fields and monitored through magnetic resonance imaging (MRI) [113]. Moreover, these nanoparticles can induce hyperthermia upon exposure to alternating magnetic fields, causing localized tumor cell death [114].

Controlled release systems aim to maintain therapeutic drug concentrations at the tumor site over extended periods, reducing systemic toxicity and improving efficacy [115]. These systems can be engineered to release their payload in response to specific stimuli within the tumor microenvironment, such as pH changes, enzymatic activity, or temperature variations [116]. Biodegradable polymers like polylactic-co-glycolic acid (PLGA) are commonly used to fabricate nanoparticles or implants that gradually degrade, releasing the encapsulated drug [117]. The Gliadel^®^ wafer is a notable example of an implantable polymeric device approved for the treatment of high-grade glioma. The device delivers carmustine directly into the resection cavity post-surgery, bypassing the BBB and minimizing systemic exposure [118]. Hydrogel-based systems offer another approach to controlled drug release [119]. Injectable hydrogels can conform to the shape of the resection cavity and provide a sustained release of therapeutics [120]. These hydrogels can be loaded with chemotherapeutic agents, growth factor inhibitors, or even nanoparticles carrying genetic material [121]. Smart delivery systems are being developed to respond dynamically to the tumor environment [122]. For instance, pH-sensitive nanoparticles can release their cargo in the acidic conditions typical of tumor tissues [123], while enzyme-responsive systems utilize enzymes overexpressed in GBM to trigger drug release [124]. These advanced delivery platforms hold promise for enhancing the specificity and effectiveness of GBM treatments.

### 3.16. Molecular Profiling and Biomarkers

Molecular profiling goes well beyond merely identifying prominent mutations, as it encompasses a detailed examination of tumors at the genomic, transcriptomic, and sometimes epigenomic levels to reveal the complex interplay of molecular alterations [16]. Through these evaluations, clinicians and researchers gain deeper insights into tumor heterogeneity by detecting subclonal populations within the same tumor mass, each potentially harboring distinct genetic or epigenetic changes that influence therapy response [16,125]. Such heterogeneity underlines the importance of high-throughput techniques, including next-generation sequencing (NGS), which facilitates the efficient and simultaneous analysis of multiple molecular aberrations [126]. NGS-based methods, combined with advanced bioinformatics pipelines, can rapidly process large datasets to pinpoint targetable mutations, overexpressed oncogenes, or inactivated tumor suppressor genes—all of which can guide therapeutic decision-making [126]. In parallel, gene expression profiling provides valuable information about aberrant signaling pathways and helps identify molecular signatures correlated with disease progression, resistance mechanisms, or sensitivity to specific agents [16,125]. This convergence of genomic and transcriptomic insights enhances the ability to select patient subgroups that share similar molecular traits and are, therefore, more likely to derive clinical benefit from targeted drugs, such as EGFR inhibitors in tumors exhibiting EGFR amplification or activating mutations [63]. Moreover, by clustering patients based on such molecular characteristics, clinical trials can be designed in a more stratified manner, thereby increasing statistical power and raising the probability of detecting true therapeutic benefits within responsive cohorts [127].

Crucially, the process of integrating these molecular techniques into routine practice involves establishing rigorous quality control measures to ensure reliable data interpretation [16]. As part of this integration, parallel assessment of copy number variations, fusion genes, and mutations in oncogenes or tumor suppressors can provide a more holistic picture of a tumor’s molecular landscape [126]. This refined stratification enables the identification of minor, but clinically significant, subpopulations that might otherwise be missed in broad, unselected patient groups [127]. Consequently, clinical trials can incorporate biomarker-driven enrichment strategies, ensuring that only individuals whose tumors harbor the relevant alterations are enrolled, thus streamlining the evaluation of novel therapies [125]. When combined with real-time molecular monitoring—whereby tumor samples can be periodically reassessed—researchers can also track the emergence of resistance mutations or changes in gene expression that may necessitate therapeutic adjustments [16,126]. Overall, the improved accuracy and scope of molecular profiling underscore its pivotal role in shaping a more personalized, and potentially more successful, approach to GBM therapy [125].

Biomarker utilization further refines this personalized strategy by distinguishing between predictive and prognostic indicators [128]. Predictive biomarkers, such as MGMT promoter methylation, specifically help determine whether a patient will benefit from a targeted or cytotoxic therapy; in the case of MGMT, methylation silences the DNA repair enzyme, thus making tumor cells more susceptible to alkylating agents like temozolomide [38]. This effect can be profound because patients with methylated MGMT often exhibit improved response rates and longer survival, underscoring the necessity of accurate methylation status testing [38]. Prognostic biomarkers, on the other hand, reveal likely disease trajectory regardless of treatment and may overlap with predictive indicators in certain contexts, highlighting the complexity of biomarker interpretation [128]. PD-L1 expression levels also serve as a pivotal predictive biomarker for immunotherapies targeting the PD-1/PD-L1 axis, although they do not guarantee therapeutic success in GBM [55]. Variations in PD-L1 expression among tumor regions and dynamic changes over time can complicate treatment decisions, underscoring the need for standardized assays and repeated measurements to refine patient selection [55].

Incorporating biomarker assessments into clinical trial frameworks is integral for capturing the full impact of novel interventions [129]. This approach allows investigators to correlate specific molecular or immunologic markers with clinical endpoints such as response rate, progression-free survival, and overall survival [129]. In turn, these correlations help define subgroup-specific benefits, guide dose optimization, and identify potential resistance mechanisms early in the drug development process [127,129]. As such, biomarker-driven strategies do not merely improve the efficiency and success rates of clinical trials—they also accelerate the transition toward a genuinely personalized treatment paradigm. By focusing on the molecular intricacies of each patient’s tumor, clinicians can adopt a more targeted selection of therapies, potentially reducing exposure to ineffectual treatments and the associated toxicities [16,125,126]. In this way, molecular profiling and the thoughtful application of both predictive and prognostic biomarkers serve as linchpins for advancing the precision medicine agenda in glioblastoma, thereby setting the stage for improved patient outcomes [129].

### 3.17. Combination Therapies

Given the complexity of GBM pathogenesis and the redundancy of signaling pathways, combination therapies targeting multiple pathways simultaneously are hypothesized to produce synergistic effects [96]. Combining agents can overcome resistance by targeting alternative pathways that tumor cells may utilize to evade single-agent therapies, enhance efficacy through simultaneous inhibition of complementary pathways, and reduce doses to minimize toxicity while maintaining efficacy [130]. Examples include the Stupp protocol, which combines temozolomide with radiotherapy to leverage the radiosensitizing effects of temozolomide [9], and trials combining EGFR inhibitors with temozolomide to block survival pathways activated by DNA damage [131]. Immunotherapy combinations, such as combining immune checkpoint inhibitors with vaccines or oncolytic viruses, may enhance immune activation against tumor cells [132]. Angiogenesis inhibitors combined with other therapies may normalize tumor vasculature, improving drug and oxygen delivery [133]. Optimizing combination regimens requires careful consideration of pharmacodynamics, potential overlapping toxicities, and scheduling to maximize synergistic effects while minimizing adverse events [134].

## 4. Challenges and Future Directions

GBM exhibits remarkable intra-tumoral heterogeneity, with distinct subpopulations of tumor cells harboring diverse genetic and epigenetic alterations that can dynamically evolve over time [17,65,125]. Single-cell sequencing studies have further underscored this complexity, revealing how coexisting subclones within a single tumor may possess varied transcriptional states and functional behaviors [17,65,125]. These divergent cellular populations can respond differently to therapeutic interventions, making it exceedingly difficult to eradicate all malignant cells with a single targeted agent. Furthermore, recent work has shown that phenotypic plasticity—wherein tumor cells shift among different lineage states—can facilitate rapid adaptation to environmental pressures, including therapy-induced stress, and drive resistance through redundant signaling pathways [65,135]. Notably, resistance does not merely stem from well-characterized genetic mutations; epigenetic modifications and tumor-stromal interactions are increasingly recognized as contributors that help malignant cells evade treatment [17,135,136,137].

Current strategies to combat therapeutic resistance involve combining multiple agents that simultaneously inhibit complementary or compensatory pathways, with the goal of mitigating the likelihood of clonal escape. Synthetic lethality approaches, which aim to exploit specific genetic or metabolic vulnerabilities in resistant cells, have begun to show early promise, particularly when supported by robust preclinical models and innovative screening platforms [65,135,136,137,138,139]. Yet, a crucial research gap remains in translating these novel combination regimens from bench to bedside. Traditional clinical trial designs may not fully capture the intricate interplay among heterogeneous tumor cell populations, underscoring the need for adaptive trial strategies that can incorporate real-time molecular monitoring and rapid regimen adjustments [65,140]. More sensitive diagnostic tools, including liquid biopsies and advanced imaging techniques, could allow clinicians to detect emerging resistant clones at earlier time points, guiding timely therapeutic interventions [65,141,142].

Despite a deeper understanding of GBM pathophysiology, meaningful extensions in patient survival remain elusive [17,143]. Newer therapies—whether molecularly targeted agents, advanced radiation modalities, or immunotherapies—can still produce off-target effects and dose-limiting toxicities, which substantially impact patients’ quality of life [143]. To overcome these persistent challenges, preclinical research has focused on leveraging combination regimens that target multiple signaling pathways and tumor-supporting microenvironmental components in parallel [144,145]. There is also a growing emphasis on personalized medicine approaches: multi-omics profiling, encompassing genomics, transcriptomics, proteomics, and emerging epigenomic and metabolomic analyses, help pinpoint precise molecular drivers in individual tumors [146]. Integrating these massive datasets with artificial intelligence (AI) and machine-learning tools has already revealed novel biomarkers and therapeutic targets that might otherwise remain undetected [147]. However, a gap remains in translating the complexity of these large-scale datasets into clinically actionable insights. Developing standardized pipelines for data processing and validation, coupled with prospective clinical trials that test AI-guided treatment decisions, represents an urgent need for the field [148].

Immunotherapies offer another promising frontier. Immune checkpoint inhibitors, chimeric antigen receptor (CAR) T cell therapies, and cancer vaccines have demonstrated the potential to elicit durable responses in some GBM patients [17]. Nevertheless, their overall efficacy is hampered by the immunosuppressive tumor microenvironment, which includes regulatory T cells, myeloid-derived suppressor cells, and immunosuppressive cytokine networks [17,149]. Overcoming these barriers may require a combination of immunotherapeutic strategies, pairing checkpoint inhibitors with novel agents that reprogram the tumor microenvironment or modulate systemic immune function [17,135,142]. Research gaps in this area include the need for more precise tools to predict which patients are most likely to benefit from immunotherapy, as well as optimized delivery methods to ensure immunotherapeutic agents achieve effective concentrations in intracranial tumor regions [65,136,150].

Lastly, advances in nanotechnology are enabling the development of nanoparticle-based systems capable of traversing the blood–brain barrier to deliver therapeutic payloads directly to tumor sites, potentially boosting drug specificity and reducing off-target toxicity [17,65,151]. Yet, major questions remain regarding the optimal size, surface chemistry, and targeting ligands that maximize intracranial uptake and therapeutic index. Clinically relevant animal models that mimic both the physiological and immunological aspects of the human brain tumor environment are urgently needed to accelerate nanoparticle innovation and ensure translational success [125,135]. These overarching research gaps, ranging from integrating multi-omics data into actionable treatment plans to refining immunotherapeutic strategies and nanoparticle design, underscore the complexity of GBM and the pressing need for multifaceted, adaptive approaches. By addressing these challenges head-on, the field stands to usher in more personalized, effective, and tolerable therapies, ultimately improving survival outcomes and quality of life for patients with this devastating disease [17,141,142,143].

## 5. Conclusions

Despite intensive efforts and technological advances, meaningful clinical breakthroughs in GBM remain elusive. Ongoing research focusing on personalized medicine, combination therapies, and emerging modalities such as immunotherapy and nanotechnology underscores the need for continued innovation. Addressing GBM’s complexity will require a multidisciplinary push to develop more effective, tolerable, and accessible treatments that finally offer patients tangible improvements in survival and quality of life.

## Figures and Tables

**Table 1:** Common genetic mutations and epigenetic modifications in GBM.

Genetic Mutation/Epigenetic Modification	Frequency in GBM (% of Cases)	Impact on Tumor Biology	Potential Approaches Under Investigation
TP53 Mutations	31–38% overall; up to 65% in secondary GBMs	Disrupts cell cycle control and apoptosis	Potential for therapies targeting p53 pathways
PTEN Mutations	24–37% (mainly in primary GBMs)	Activates PI3K/Akt signaling, promoting proliferation and survival	Use of PI3K/Akt pathway inhibitors
EGFR Amplification and Mutations	36–60% in primary GBMs; EGFRvIII in 20–50% of amplified cases	Enhances cell growth via receptor activation	EGFR inhibitors and antibodies targeting EGFRvIII variant
NF1 Mutations or Deletions	15–17%	Affects RAS/MAPK signaling pathways	Therapies targeting RAS/MAPK components
PIK3CA and PIK3R1 Mutations	PIK3CA: 7–10%; PIK3R1: 7–8%	Activates PI3K/Akt pathway	PI3K inhibitors
RB1 Mutations	8–13%	Impairs cell cycle regulation via retinoblastoma pathway	CDK inhibitors targeting cell cycle dysregulation
CDKN2A Deletion (p16^INK4^) and p14^ARF^)	31–78% in primary GBMs	Loss of cell cycle inhibition, increased proliferation	CDK4/6 inhibitors; restoring cell cycle checkpoints
ATRX Mutations	Common in secondary GBMs and lower-grade gliomas	Involved in telomere maintenance	Targeting telomere elongation mechanisms
TERT Promoter Mutations	58% in primary; 28% in secondary GBMs	Increases telomerase activity (anti-senescence),	Telomerase inhibitors
MGMT Promoter Hypermethylation	36% in primary; 75% in secondary GBMs	Reduces DNA repair capacity; better response to alkylating agents	Predictive biomarker for temozolomide efficacy
Hypermethylation of Tumor Suppressor Genes	RB1: 14% primary, 43% secondary; CDKN2A-p14^ARF^: 6% primary, 31% secondary	Silencing of genes critical for cell cycle and apoptosis	Use of demethylating agents to reactivate tumor suppressor genes
Loss of Heterozygosity (LOH) on Chromosome 10	Up to 70% in primary GBMs	Associated with PTEN loss; contributes to tumor progression	Important to target PTEN pathway
Chromosome 9p21 Deletion	31–78% in primary GBMs	Loss of CDKN2A locus, leading to cell cycle dysregulation	Need for therapies targeting cell cycle control

**Table 2:** Key signaling pathways in GBM and potential therapeutic targets.

Signaling Pathway	Key Components	Role in GBM Progression	Potential Targeted Therapies
p53 Pathway	TP53 gene, MDM2, p21	Regulates cell cycle and apoptosis; mutations lead to uncontrolled cell proliferation and impaired cell death	MDM2 inhibitors (e.g., RG7112), compounds restoring p53 function (e.g., PRIMA-1)
PI3K/AKT/mTOR Pathway	PI3K (PIK3CA), AKT, mTOR, PTEN	Promotes cellular growth, survival, and metabolism; frequently activated due to PTEN loss or PIK3CA mutations	PI3K inhibitors (e.g., BKM120), AKT inhibitors (e.g., perifosine), mTOR inhibitors (e.g., everolimus)
EGFR Pathway	EGFR, EGFRvIII mutant, downstream effectors (RAS, AKT)	Enhances tumor cell proliferation and survival; EGFR amplification/mutation leads to constitutive activation	EGFR tyrosine kinase inhibitors (e.g., erlotinib), monoclonal antibodies, vaccines targeting EGFRvIII
NF-κB Pathway	NF-κB proteins (p65, p50), IκB kinase (IKK) complex	Drives inflammation, promotes tumor growth and resistance to apoptosis	NF-κB inhibitors (e.g., parthenolide, BAY 11-7082)
Wnt Signaling Pathway	Wnt ligands, frizzled receptors, β-catenin	Regulates cell proliferation and differentiation; aberrant activation contributes to tumor aggressiveness	Wnt pathway inhibitors (under investigation)
TERT Pathway	Telomerase reverse transcriptase (TERT)	Maintains telomere length, allowing unlimited cell division	Telomerase inhibitors, TERT-targeted therapies
CDKN2A/pRB Pathway	CDKN2A gene (p16^INK4A^, p14^ARF^), RB1 protein	Controls cell cycle progression; loss leads to unchecked proliferation	CDK4/6 inhibitors (e.g., palbociclib), strategies to restore pathway function
c-Met Pathway	c-Met receptor, hepatocyte growth factor (HGF)	Promotes cell growth, invasion, and angiogenesis	c-Met inhibitors (e.g., crizotinib, cabozantinib), monoclonal antibodies (e.g., onartuzumab)
FGFR Pathway	FGFR receptors, FGF ligands	Involved in cell proliferation and survival; less commonly altered in GBM	FGFR inhibitors (e.g., futibatinib, pemigatinib)
BRAF Pathway	BRAF kinase (V600E mutation)	Activates MAPK/ERK pathway, promoting growth	BRAF inhibitors (e.g., dabrafenib, vemurafenib)
Src Pathway	Src family kinases	Facilitates proliferation and invasion	Src inhibitors (e.g., dasatinib)
RAS/MAPK Pathway	RAS proteins, RAF, MEK, ERK	Controls cell proliferation and differentiation; overactivation leads to tumor growth	MEK inhibitors, oncolytic viruses targeting RAS pathway
MGMT	O6-Methylguanine-DNA methyltransferase	Repairs DNA damage from alkylating agents	MGMT inhibitors
VEGF Signaling	Vascular endothelial growth factor (VEGF), VEGF receptors	Stimulates angiogenesis, supporting tumor vascularization	Anti-VEGF therapies (e.g., bevacizumab)
TGF-β Pathway	Transforming growth factor-beta (TGF-β)	Promotes invasion and immunosuppression	TGF-β inhibitors (e.g., galunisertib)
HDAC Pathway	Histone deacetylases	Epigenetic regulation; overactivity leads to aberrant gene expression	HDAC inhibitors (e.g., vorinostat, panobinostat)
Notch Pathway	Receptors (Notch1–4), ligands (Dll1, Dll3, Dll4, Jagged1–2), γ-secretase, RBPJK	Maintains GSCs, promotes treatment resistance, drives tumor growth, angiogenesis, and stemness under hypoxia	GSIs (DAPT, RO4929097), ASIs (INCB3619), miRNAs (miR-34a, miR-181c), arsenic trioxide, tipifarnib, CB-103
Hedgehog Pathway	Sonic Hedgehog (SHH), patched (PTCH1/2), smoothened (SMO), GLI1/2/3	Regulates tumor growth, stem cell maintenance, drug resistance, and promotes angiogenesis and invasion	SMO inhibitors (e.g., vismodegib, sonidegib), GLI inhibitors (e.g., GANT-61), combination therapies to overcome resistance
MAPK Pathway	EGFR, PDGFRA, BRAF, MAPK	Promotes cell proliferation, survival, and therapy resistance via pathway hyperactivation (high MAPK activity correlates with poor survival and increased tumor aggressiveness)	MAPK inhibitors (e.g., BRAF inhibitors); potential for combination therapies targeting MAPK and PI3K/AKT pathways

**Table 3:** (**a**) EGFR inhibitors for GBM. (**b**) Other receptor tyrosine kinase (RTK) inhibitors for GBM. (**c**) Cell cycle (CDK4/6) inhibitors for GBM. (**d**) MET/ALK/multiple RTK inhibitors for GBM.

(a)			
Agent	Mechanism	Clinical Phase/Population	Findings
**Gefitinib**	First-generation EGFR tyrosine kinase inhibitor (TKI)	Phase II (recurrent GBM) (e.g., NCT01520870)	- Poor BBB penetration - EGFR alterations in GBM are heterogeneous; not all tumors rely on EGFR signaling
**Dacomitinib**	Pan-EGFR TKI (inhibits EGFR, HER2, HER4)	Phase II (recurrent GBM) (e.g., NCT02447419)	- Still challenged by BBB penetration - Broader than gefitinib, but GBM evolves alternate pathways
**Osimertinib**	Third-generation EGFR TKI, better BBB permeability	Early-phase/preclinical (recurrent GBM)	- Promising in preclinical models due to improved BBB penetration - Further phase I/II trials needed to determine safety and efficacy
**Nimotuzumab**	Anti-EGFR monoclonal antibody (mAb)	Phase II/III (various GBM populations)	- Mixed results: some modest improvements in specific subgroups - Reduced toxicity vs. other anti-EGFR mAbs because of intermediate affinity
**Depatux-M (ABT-414)**	Antibody–drug conjugate targeting EGFR; delivers cytotoxic agent	Phase II/III (EGFR-amplified GBM) (e.g., NCT02573324)	- Some efficacy in EGFR-amplified GBM - Ocular toxicity reported; highlights the need for careful dosing and patient selection
**Challenges**: Poor BBB penetration. Intratumoral heterogeneity and EGFR pathway redundancy. Adaptive resistance (GBM cells switch to alternative pathways).			
**(b)**			
**Agent**	**Mechanism**	**Clinical Phase/Population**	**Findings**
**Cabozantinib**	Inhibits MET and VEGFR2 (angiogenesis)	Phase II (recurrent GBM) (e.g., NCT00704288)	- Modest activity in heavily pretreated patients - Notable toxicities (hypertension, fatigue, etc.)
**Capmatinib (INC280)**	Selective MET inhibitor	Phase II (recurrent GBM) (e.g., NCT01870726)	- Limited efficacy overall - Possible benefit in tumors with MET amplification or alterations
**Erdafitinib**	Pan-FGFR inhibitor (incl. FGFR3-TACC3 fusions)	Phase II (recurrent GBM) (e.g., NCT01703481)	- Partial responses in some patients with FGFR alterations - Ongoing trials with biomarker selection
**Challenges**: Overlapping growth pathways (GBM can activate PI3K, PDGF, or EGFR). BBB penetration and systemic toxicity. Small subsets of GBM harbor these specific driver alterations.			
**(c)**			
**Agent**	**Mechanism**	**Clinical Phase/Population**	**Findings**
**Palbociclib**	CDK4/6 inhibitor; blocks G1 → S phase transition	Phase II (recurrent GBM) (e.g., NCT01227434)	- No significant efficacy as monotherapy - Ongoing combinations with radiation or targeted agents
**Ribociclib**	CDK4/6 inhibitor	Phase I/II (recurrent GBM) (e.g., NCT02345824)	- Limited single-agent benefit - Potential synergy with other pathways (e.g., mTOR inhibitors)
**Challenges**: GBM often has multiple genetic alterations (RB, p53, PTEN), so simply blocking CDK4/6 is not enough. Tumors may develop resistance to CDK4/6 inhibitors, reducing their effectiveness over time. Identifying patients who would benefit most from these therapies is challenging due to the lack of reliable biomarkers.			
**(d)**			
**Agent**	**Mechanism**	**Clinical Phase/Population**	**Findings**
**Bortezomib/Marizomib**	Proteasome inhibitors (alter proteostasis)	Bortezomib: phase I/II; marizomib: phase III (e.g., NCT03345095)	- Bortezomib limited by BBB and toxicity - Marizomib under combination of trials (TMZ + RT), aiming for synergy
**Bevacizumab**	Anti-VEGF mAb (angiogenesis blockade)	Approved for Recurrent GBM	- Improves progression-free survival, less proven benefit in overall survival - Combined with chemo or RT
**Challenges**: Many agents, like bortezomib, face difficulty effectively reaching brain tumor sites due to the BBB. Agents such as bortezomib are limited by systemic toxicity, reducing their feasibility for long-term use or high dosing. Bevacizumab shows improved progression-free survival, but limited evidence of extending overall survival in patients.			

**Table 4:** (**a**): CAR T cells for GBM. (**b**) Vaccines under investigation for GBM.

(a)			
Agent	Target	Clinical Phase/Population	Key Findings and Rationale
**EGFRvIII-targeted CAR T Cells**	EGFRvIII mutation (common in GBM)	Early-phase (e.g., NCT02209376)	- Safe but limited efficacy due to antigen loss and immunosuppressive microenvironment
**IL13Rα2-targeted CAR T Cells**	IL13Rα2 (overexpressed in GBM)	Phase I (case reports)	- Dramatic regression in a single case report - Studies ongoing to confirm broad efficacy and overcome tumor heterogeneity
**HER2-targeted CAR T Cells**	HER2 receptor	Early-phase	- Preliminary safety established; potential synergy with other immunotherapies
**Challenges**: GBM’s antigen heterogeneity (tumors can downregulate the target). T cell trafficking into the brain. Immunosuppressive environment (TAMs, MDSCs, Tregs).			
**(b)**			
**Agent**	**Mechanism**	**Clinical Phase/Population**	**Key Findings and Rationale**
**Rindopepimut**	Peptide vaccine targeting EGFRvIII	Phase III (ACT IV; NCT01480479)	- Did not improve OS vs. control - Trial halted; underscores how GBM escapes single-target therapies
**DCVax^®^-L**	Dendritic cell vaccine with autologous tumor lysate	Phase III (NCT00045968)	- Interim data suggest possible survival benefit - Full results pending; likely works best in low tumor burden
**Challenges**: Antigen loss/tumor heterogeneity. GBM’s robust immune evasion mechanisms.			

**Table 5:** Oncolytic viruses for GBM.

Agent	Virus Type/Target	Clinical Phase/Population	Key Findings and Rationale
**PVSRIPO**	Engineered poliovirus targeting CD155	Phase I/II (recurrent GBM)	- Demonstrated safety; some patients have prolonged survival - Requires strong anti-tumor immune response
**DNX-2401**	Oncolytic adenovirus selectively replicating in GBM	Phase I (recurrent GBM) (NCT00805376)	- Induces immune response; some durable remissions - Combining with other immunotherapies is under investigation
**G47Δ**	Genetically engineered herpes simplex virus	Phase II (Japan)	- Conditional approval in Japan for recurrent GBM - Showed improved survival vs. historical controls
**Challenges**: Achieving uniform virus distribution in a large, heterogeneous tumor. Success depends heavily on eliciting a strong and targeted anti-tumor immune response, which can vary significantly between patients.			

**Table 6:** Epigenetic modulators for GBM.

Agent	Mechanism	Clinical Phase/Population	Key Findings and Rationale
**Vorinostat**	HDAC inhibitor; alters gene expression, induces apoptosis	Phase II	- Limited efficacy as monotherapy - Combining with RT or chemotherapy being explored
**Azacitidine**	DNMT inhibitor; demethylates DNA to restore tumor suppressor genes	Phase II (NCT03666559)	- Ongoing; rationale is that epigenetic changes in GBM may re-sensitize to therapy
**Challenges**: Both HDAC and DNMT inhibitors often show limited effectiveness as standalone treatments. Tumor cells can develop resistance to these agents, limiting their long-term utility. Many GBMs show epigenetic dysregulation. Reversing some of these changes might re-open tumor sensitivity to immunotherapy or chemo.			

## Data Availability

No new data were created.
